# Impact of echinocandins as first-choice therapy in cardiothoracic high-risk patients with candidemia

**DOI:** 10.1186/cc12023

**Published:** 2013-03-19

**Authors:** G Langebartels, Y Choi, J Catteleans, T Wahlers

**Affiliations:** 1 University of Cologne, Germany

## Introduction

Invasive candidemia is a major cause of increased mortality among ICU patients. Antifungal agents like liposomale amphotericin B and azoles could not accomplish the claim to be first choice in the treatment of invasive fungal infection (IFI) because of side effects and effectiveness. Especially, cardiothoracic surgery patients as a group of high-risk patients are in a focus for new strategies and agents. A new class of antimycotic agents, the echinocandins, with a low profile of side effects, low interactive potential and high effectiveness in the treatment of candidemia, is a powerful option in the treatment of IFI. We report our single-center experience with a modified clinical treatment approach based on clinical score of Leon and using echinocandins as first-line therapy for proven and suspected fungal infection.

## Methods

**Figure 1:**
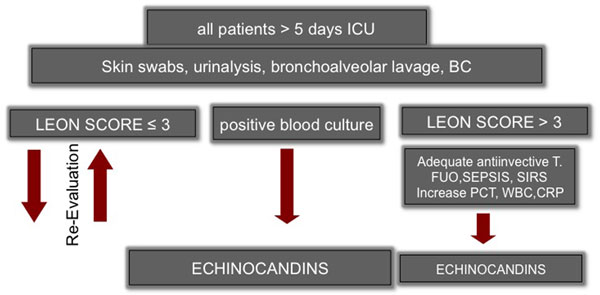
**Cologne antifungal strategy**.

From May 2011 to October 2012, 2,844 patients were treated on our cardiothoracic ICU. We evaluated 37 cardiothoracic postoperative patients with proven or suspected IFI or prophylaxis (Figure 1). The records were evaluated for cardiothoracic procedures, microbiological and yeast date, cardiothoracic surgery score (CASUS), ICU and clinical data.

## Results

Mean age was 67.4 years with 64% male patients. Most patients had combined CABG and valve procedure (*n *= 20), other groups were HTX and LTX (*n *= 4), assist therapy (*n *= 4), TAVI (*n *= 3) and other procedures. Mean predicted mortality using the logarithmic CASUS score at the onset of IFI was 59%. *C. albicans *was isolated in 73%, *C. glabrata *in 21%. Length of antifungal treatment using micafungin in 30 cases was 14 ± 5 days. Eradication of yeast was successful in 79% but mortality of all patients remains high at 36.8% but was lower than predicted in the CASUS score. Mortality was not yeast related.

## Conclusion

Our described treatment approach shows encouraging results for the treatment of IFI especially in high-risk cardiothoracic patients.
